# Role of the Contra-Lesional Corticoreticular Tract in Motor Recovery of the Paretic Leg in Stroke: A Mini-Narrative Review

**DOI:** 10.3389/fnhum.2022.896367

**Published:** 2022-05-26

**Authors:** Sung Ho Jang, Min Jye Cho

**Affiliations:** Department of Physical Medicine and Rehabilitation, College of Medicine, Yeungnam University, Taegu, South Korea

**Keywords:** corticoreticulospinal tract, corticoreticular tract, motor recovery, stroke, diffusion tensor tractography

## Abstract

This review discusses the role of the contra-lesional corticoreticular tract (CRT) in motor recovery of the paretic leg in stroke patients by reviewing related diffusion tensor tractography studies. These studies suggest that the contra-lesional CRT can contribute to the motor recovery of the paretic leg in stroke patients, particularly in patients with complete injuries of the ipsilesional corticospinal tract and CRT. Furthermore, a review study reported that the motor recovery of the paretic ankle dorsiflexor, which is mandatory for achieving a good gait pattern without braces in hemiparetic stroke patients, was closely related to the contra-lesional CRT. These results could be clinically important in neuro-rehabilitation. For example, the contra-lesional CRT could be a target for neuromodulation therapies in patients with complete injuries of the ipsilesional corticospinal tract and CRT. On the other hand, only three studies were reviewed in this review and one was a case report. Although the CRT has been suggested to be one of the ipsilateral motor pathways from the contra-lesional cerebral cortex to the paretic limbs in stroke, the role of the CRT has not been elucidated clearly. Therefore, further prospective follow-up studies combining functional neuroimaging and transcranial magnetic stimulation for the paretic leg with diffusion tensor tractography will be useful for elucidating the role of the contra-lesional CRT in stroke patients.

## Introduction

The motor system is classified grossly into pyramidal and extrapyramidal systems in the human brain (Nathan and Smith, 1955; Mendoza and Foundas, 2007). The corticoreticulospinal tract (CRST) is a representative neural tract for the motor function in the extrapyramidal system (Nathan and Smith, 1955; Matsuyama et al., 2004; Mendoza and Foundas, 2007; Yeo et al., 2012; Jang and Lee, 2019). The CRST consists of the corticoreticular tract (pathway) (CRT) and reticulospinal tract (Nathan and Smith, 1955; Matsuyama et al., 2004; Mendoza and Foundas, 2007; Yeo et al., 2012; Jang and Lee, 2019). The CRT was reported to originate mainly from the premotor cortex and terminate at the pontomedullary reticular formation (Matsuyama et al., 2004; Mendoza and Foundas, 2007; Yeo et al., 2012; Jang and Lee, 2019). The reticulospinal tract, which originates from the pontomedullary reticular formation, is known to descend bilaterally (Matsuyama et al., 2004; Mendoza and Foundas, 2007; Yeo et al., 2012; Jang and Lee, 2019). The CRST innervates the axial muscles and the proximal muscles of the extremities: ~30–40% of the total motor function of the proximal muscles (Demeurisse et al., 1980; Paternostro-Sluga et al., 2008; Yeo et al., 2013; Kwon and Jang, 2014; Yoo et al., 2014). As a result, functionally, the CRST is involved in gait and postural control (Kably and Drew, 1998; Matsuyama et al., 2004; Mendoza and Foundas, 2007). The motor function of the animals depends on the extrapyramidal system, including the reticulospinal tract. In contrast, the corticospinal tract is the major neural tract for the motor function in humans (Rothwell, 2006). Previous studies suggested that the CRST is important in the motor function, next to the corticospinal tract, in the human brain (Nathan and Smith, 1955; Do et al., 2013; Jang, 2014; Lee and Jang, 2015; Jang and Lee, 2019). Therefore, detailed knowledge of the CRST is clinically important, particularly neuro-rehabilitation (Li, 2017; O'Brien et al., 2018,O'Leary et al., 2021).

Little is known about the CRST in the human brain, and it appears to be related to the limitations of the tools for evaluating the CRST (Benecke et al., 1991; Miyai et al., 2001, 2002; Yeo et al., 2013; Jang et al., 2017). Functional neuroimaging techniques and transcranial magnetic stimulation are used mainly to investigate the CRST (Benecke et al., 1991; Miyai et al., 2001, 2002; Yeo et al., 2013; Jang et al., 2017). On the other hand, these evaluation tools have a significant limitation in that they do not allow anatomical visualization of the CRST in the live human brain. By contrast, diffusion tensor tractography (DTT), which is reconstructed based on diffusion tensor imaging (DTI) data, allows for an anatomical reconstruction and evaluation of the CRT three-dimensionally in the live human brain (Yeo et al., 2012). The unique advantages of DTT are that the characteristics of the entire CRT can be estimated in terms of the anatomical configuration and various DTT parameters, including fractional anisotropy (FA: state of white matter organization as it is a measure of the degree of directionality and integrity of white matter microstructures), apparent diffusion coefficient (ADC; the magnitude of water diffusion), and tract volume (or fiber number: the number of voxels within a neural tract, which represents the number of fibers within of a neural tract) (Mori et al., 1999; Assaf and Pasternak, 2008; Neil, 2008). As a result, many DTT-based studies have reported the anatomy, function, prognosis prediction, and neural plasticity of the CRT (Jang et al., 2013; Yeo and Jang, 2013; Jang and Seo, 2014; Jang and Yeo, 2014; Jang and Lee, 2016; Soulard et al., 2020).

Detailed information on the recovery mechanisms for motor function in stroke patients is essential for establishing effective neuro-rehabilitation strategies. After introducing DTI, a few recovery mechanisms of injured CRT in stroke patients have been reported: recovery along its original anatomical pathway, recovery through peri-lesional reorganization, and recovery through the transcallosal pathway (Yeo and Jang, 2013; Jang and Yeo, 2014; Jang et al., 2015, 2018; Jang and Chang, 2016; Jang and Lee, 2016). On the other hand, a few DTT-based studies reported that the motor recovery of the paretic leg was related to the contribution of the contra-lesional CRT in stroke patients (Jang et al., 2013; Jang and Lee, 2017; Jang and Cho, 2022). These results could be clinically important in neuro-rehabilitation, particularly in terms of non-invasive neuromodulation. This review discusses the role of the contra-lesional CRT in motor recovery of the paretic leg in stroke patients by reviewing DTT studies.

## Methods

Studies that investigated the state of the contra-lesional CRT related to the motor recovery of the paretic leg in stroke patients using DTT were searched. Relevant studies published until March 10, 2022, were searched using the following electronic databases: Google Scholar and MEDLINE database (PubMed). The keywords/abbreviations for identifying potentially relevant articles were DTI, DTT, CRT, transcranial magnetic stimulation, motor recovery, leg, stroke, hemorrhage, and infarction. This review was limited to studies involving humans with stroke. Overall, three studies were selected and reviewed (Jang et al., 2013; Jang and Lee, 2017; Jang and Cho, 2022).

### Diffusion Tensor Tractography Studies on the Role of the Contra-Lesional Corticoreticular Tract in Motor Recovery of the Paretic Leg in Stroke

In 2013, Jang et al. examined the role of the CRT in relation to the walking ability and motor function in chronic hemiparetic stroke patients (Jang et al., 2013) (Table 1). The authors recruited 54 hemiparetic stroke patients with a supratentorial lesion (hemorrhage or infarction) who showed a complete ipsilesional corticospinal tract injury (discontinuation of the corticospinal tract around or below the lesion on DTT) and more than 3 months had passed after stroke onset. The fiber volume of the contra-lesional CRT in the patients who could walk independently was significantly higher than those of the patients who could not walk and normal control subjects. The fiber volume of the contra-lesional CRT had a moderate positive correlation with the walking ability and a mild positive correlation with the motor function of the paretic arm and leg. On the other hand, the FA value and fiber volume of the contra-lesional corticospinal tract (CST) did not correlate with the walking ability and motor function of the paretic arm and leg. These results suggest that the increased neural fiber number of the contra-lesional CRT was closely related to the walking ability and motor function of the paretic leg in chronic hemiparetic stroke patients. As a result, the authors concluded that the compensation (increased neural fiber number) of the contra-lesional CRT appeared to be one of the recovery mechanisms of walking ability and motor recovery mechanisms of the paretic leg after stroke (Jang et al., 2013). The advantage of this study is that three kinds of DTT parameters (FA, ADC, fiber number) were analyzed for the CRT and CST. Another advantage was that the authors examined the effect of the CRT in the condition of complete injury of the ipsilesional corticospinal tract. On the other hand, they did not control the effect of the ipsilesional CRT because 7.4% of patients showed intact integrity of the ipsilesional CRT (Jang et al., 2013).

**Table 1:** Diffusion tensor tractography studies on the role of the contra-lesional corticoreticular tract in the motor recovery of the paretic leg in stroke patients.

**References**	**Number of subjects**	**Brain pathology**	**Analyzed neural tract**	**DTT analysis method**
Jang et al. (2013)	54	ICH: 39 patients	CRT	DTT parameter
		Infarction: 15 patients	CST	
Jang and Lee (2017)	1	ICH	CRT	Configuration
			CST	
Jang and Cho (2022)	36	ICH: 16 patients	CRT	DTT parameter
		Infarction: 20 patients	CST	

*DTT, diffusion tensor tractography; ICH, intracerebral hemorrhage; CRT, corticoreticular tract; CST, corticospinal tract*.

**Figure 1:**
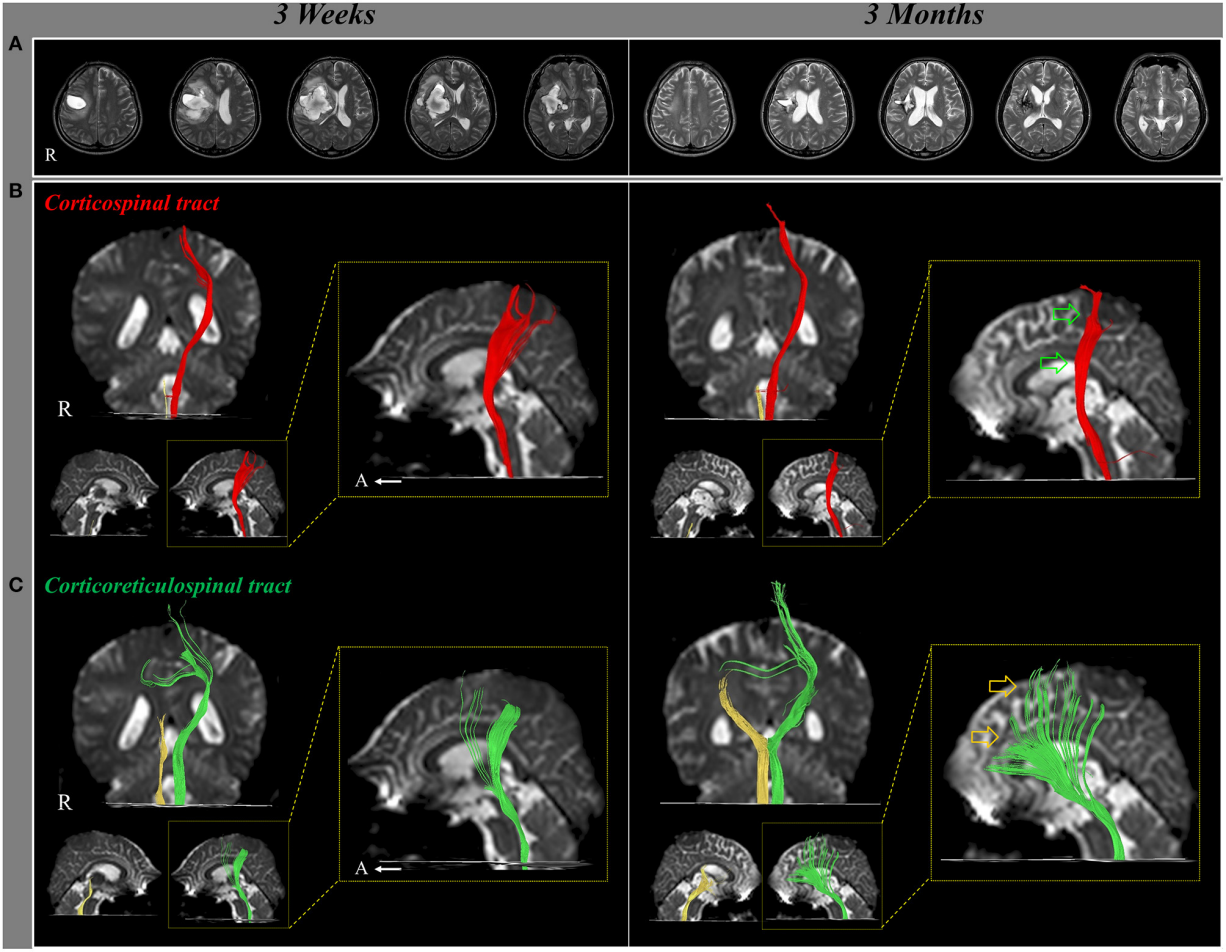
Changes of the neural tracts during the motor recovery of the paretic (left) leg. **(A)** T2-weighted MR images show a putaminal hemorrhage in the right hemisphere at 3 weeks and 3 months after onset. **(B)** Result of diffusion tensor tractography (DTT), the right corticospinal tract (CST) shows discontinuation at the brainstem on both 3-week and 3-month DTT. By contrast, on 3-month DTT, the left CST has become thinner (green arrow) compared with 3-week DTT. **(C)** The right corticoreticulospinal tract (CRT) shows discontinuation below the lesion on both 3-week and 3-month DTT. On 3-month DTT, the left CRT has become thicker (yellow arrow) compared with 3-week DTT (reprinted with permission from Jang and Lee, 2017).

In 2017, Jang and Lee reported a patient who revealed motor recovery of the paretic leg by activating the contra-lesional CRT, which was demonstrated follow-up DTTs (Jang and Lee, 2017). A 50-year-old male patient presented with complete paralysis of the left arm and leg at the onset of a right putaminal hemorrhage. The patient underwent comprehensive rehabilitative therapy from 3 weeks to 3 months after onset. During ~2 month's rehabilitation, his left hemiparesis had recovered gradually [Medical Research Council (MRC), shoulder abductor; 0–>2^+^, elbow flexor: 0–>4^−^, finger extensor; 0–>0, hip flexor; 1–>4^−^, knee extensor; 3–>4^+^ and ankle dorsiflexor; 2^−^->2^−^]. As a result, he could walk independently at 3 months after onset. The contralesional CRT revealed discontinuation at the subcortical white matter level on 3-week DTT, but it became thicker with the recovery of integrity to the cerebral cortex level on 3-month DTT. By contrast, the discontinued integrities of the other neural tracts (ipsilesional and contra-lesional corticospinal tract, and ipsilesional CRT) did not show significant changes on 3-week and 3-month DTTs. Consequently, the authors concluded that the motor recovery of the proximal muscles of the paretic arm and leg were due mainly to the activation of the contra-lesional CRT. The advantage of this study was that the authors demonstrated that the contra-lesional CRT was responsible for the motor recovery of the paretic arm and leg using follow-up DTTs from the early to chronic stages of stroke; however, this was a single case report. Furthermore, the authors provided only configurational changes without DTT parameters (Jang and Lee, 2017) (Figure 1).

Recently, Jang and Cho (2022) reported that the contra-lesional CRT and corticospinal tract were closely related to the motor recovery of the paretic ankle dorsiflexor. Thirty-six chronic hemiparetic stroke patients with supratentorial lesions (hemorrhage: 16 patients and infarction: 20 patients) who exhibited severe motor weakness of the contra-lesional ankle dorsiflexor (MRC score at onset of 0–1), complete injuries of the ipsilesional CST and CRT [complete discontinuation of the ipsilesional CRT and CRT around or below the lesion on DTT at a chronic stage (more than 3 months after stroke onset)] and underwent comprehensive rehabilitation until the chronic stage. Twenty-one (58.3%) of the 36 patients revealed good recovery (chronic stage MRC ≥ 2) of the contra-lesional ankle dorsiflexor (good recovery group), whereas 15 patients (41.7%) showed poor recovery (chronic stage MRC < 2, poor recovery group). The tract volume of the contra-lesional CRT in the good recovery group was higher than that of the poor recovery group. A strong positive correlation was found between the tract volume of the contra-lesional CRT and the MRC score for the contra-lesional ankle dorsiflexor. A moderate positive correlation was detected between the tract volume of the contra-lesional corticospinal tract and the MRC score of the contra-lesional ankle dorsiflexor. As a result, the authors concluded that the number of neural fibers of the contra-lesional CRT and corticospinal tract was closely related to the recovery of contra-lesional ankle dorsiflexor in stroke patients with complete injuries of the ipsilesional CRT and corticospinal tract. On the other hand, the contra-lesional CRT was more closely related than the contra-lesional corticospinal tract. The advantage of this study was that the authors demonstrated the role of the contralesional CRT and CST by recruiting only patients with complete injuries to the ipsilesional CRT and corticospinal tract. Another advantage of this study was that the authors excluded the effect of rehabilitation by recruiting only patients who underwent comprehensive rehabilitation until the chronic stage. The limitations of this study were that this study did not include the motor function of other joint muscles except for the ankle dorsiflexor.

### Transcranial Magnetic Stimulation Evidence for the Contra-Lesional Corticoreticular Tract in Stroke Patients

The ipsilateral motor pathway from the contra-lesional cerebral cortex to the paretic limbs is recognized as a motor recovery mechanism in stroke patients (Wassermann et al., 1991; Turton et al., 1996; Netz et al., 1997; Ziemann et al., 1999; Kim et al., 2004; Jang, 2009; Cleland and Madhavan, 2021b; Cleland et al., 2021). Many studies have reported the identity of the ipsilateral motor pathway based on the characteristics of the motor evoked potential obtained at the ipsilateral paretic limb muscles in stroke patients (Turton et al., 1996; Netz et al., 1997; Ziemann et al., 1999; Kim et al., 2004; Cleland and Madhavan, 2021b). The common characteristics of the motor evoked potentials of the ipsilateral motor pathway were the higher excitatory threshold (require more temporal summation for excitation of spinal motor neurons), delayed latency (slow conduction velocity due to smaller fibers and synaptic delays), and smaller amplitude (smaller fibers) compared to those of the contra-lesional corticospinal tract (Rossini et al., 1994; Turton et al., 1996; Netz et al., 1997; Ziemann et al., 1999; Kim et al., 2004). Many neural tracts for the motor function, including the uncrossed corticospinal tract and extrapyramidal tracts (e.g., the CRST), have been suggested as the ipsilateral motor pathway (Cleland and Madhavan, 2021b). The wide range of delayed latency (5–14 ms) on motor evoked potentials obtained at the paretic or normal ipsilateral limb muscles have been reported (Wassermann et al., 1991; Turton et al., 1996; Netz et al., 1997; Ziemann et al., 1999; Kim et al., 2004; Forrester et al., 2006; Sivaramakrishnan and Madhavan, 2020; Cleland et al., 2021). The ipsilateral motor pathway can be classified grossly into two groups based on the delayed latency: shorter delayed (5–6 ms) and longer delayed (12–14 ms) (Wassermann et al., 1991; Turton et al., 1996; Netz et al., 1997; Ziemann et al., 1999; Kim et al., 2004; Forrester et al., 2006; Sivaramakrishnan and Madhavan, 2020; Cleland et al., 2021). Muller et al. suggested that an ipsilateral motor pathway showing shorter delayed latency (5–6 ms) appeared to be the uncrossed corticospinal tract that was attributed to the decrease in interhemispheric transcallosal inhibition from the ipsilesional side toward the contralesional side after a brain injury (Netz et al., 1997; Ziemann et al., 1999; Kim et al., 2004). On the other hand, a longer delayed latency (12–14 ms) was indicated by the other neural tracts for the motor function in the extrapyramidal system, such as the CRST (Wassermann et al., 1991; Turton et al., 1996; Cleland et al., 2021). Some studies reported that these motor evoked potentials with delayed latency were evoked more easily at the proximal arm muscles than the distal arm muscles and from the premotor cortex than the primary motor cortex, suggesting the possibility of the CRST (Turton et al., 1996; Muller et al., 1997; Alagona et al., 2001). On the other hand, the identity of the ipsilateral motor pathway in stroke patients has not been elucidated clearly thus far, and there is some controversy (Jang, 2009; Cleland and Madhavan, 2021b). On the other hand, the majority of transcranial magnetic stimulation studies on the ipsilateral motor pathway were performed for the arm muscles, whereas much fewer studies reported the ipsilateral motor evoked potentials of the paretic leg (Benecke et al., 1991; Wassermann et al., 1991; Turton et al., 1996; Netz et al., 1997; Ziemann et al., 1999; Kim et al., 2004; Forrester et al., 2006; Madhavan et al., 2010; Jayaram et al., 2012; Tan et al., 2016; Tan and Dhaher, 2017; Sivaramakrishnan and Madhavan, 2020; Cleland and Madhavan, 2021a,b; Cleland et al., 2021). As a result, further prospective follow-up studies combining functional neuroimaging and transcranial magnetic stimulations for the paretic leg with DTT would be useful for elucidating the identity of the ipsilateral motor pathway in stroke patients.

## Conclusions

This review discussed the role of the contra-lesional CRT in motor recovery of the paretic leg in stroke patients by reviewing three DTT-based studies (Jang et al., 2013; Jang and Lee, 2017; Jang and Cho, 2022). Overall, the contra-lesional CRT can contribute to the motor recovery of the paretic leg in stroke patients, particularly in patients with complete injuries of the ipsilesional corticospinal tract and CRT (Jang et al., 2013; Jang and Lee, 2017; Jang and Cho, 2022). Furthermore, one study reported that the motor recovery of the paretic ankle dorsiflexor, which is mandatory for achieving a good gait pattern without braces in hemiparetic stroke patients, was closely related to the contra-lesional CRT (Jang and Cho, 2022). These results could be clinically important in neuro-rehabilitation. For example, neuromodulation therapies, such as repetitive transcranial magnetic stimulation and transcranial direct current stimulation, could be applied to the contra-lesional CRT for motor recovery of the paretic leg when a patient shows complete injuries to the ipsilesional corticospinal tract and CRT (O'Brien et al., 2018; O'Leary et al., 2021). On the other hand, only three studies were reviewed, and one was a case report. In addition, although the CRST has been suggested as one of the ipsilateral motor pathways from the contra-lesional cerebral cortex to the paretic limbs in stroke, the role of the CRST has not been elucidated clearly (Cleland and Madhavan, 2021b). Therefore, further prospective follow-up studies combining functional neuroimaging and transcranial magnetic stimulation for the paretic leg with DTT will be needed to determine the role of the contra-lesional CRST as an ipsilateral motor pathway in stroke patients.

## Author Contributions

SJ and MC contributed to conception, design of the study, wrote the first draft of the manuscript, and wrote sections of the manuscript. MC organized the database and performed the statistical analysis. All authors contributed to manuscript revision, read, and approved the submitted version.

## Funding

This work was supported by the National Research Foundation of Korea (NRF) Grant funded by the Korean Government (MSIP) (No. 2021R1A2B5B01001386).

## Conflict of Interest

The authors declare that the research was conducted in the absence of any commercial or financial relationships that could be construed as a potential conflict of interest.

## Publisher's Note

All claims expressed in this article are solely those of the authors and do not necessarily represent those of their affiliated organizations, or those of the publisher, the editors and the reviewers. Any product that may be evaluated in this article, or claim that may be made by its manufacturer, is not guaranteed or endorsed by the publisher.

## Abbreviations

CRST: Corticoreticulospinal tract
CRT: Corticoreticular tract
DTT: Diffusion tensor tractography
DTI: Diffusion tensor imaging
FA: Fractional anisotropy
ADC: Apparent diffusion coefficient
CST: Corticospinal tract
MRC: Medical Research Council.
